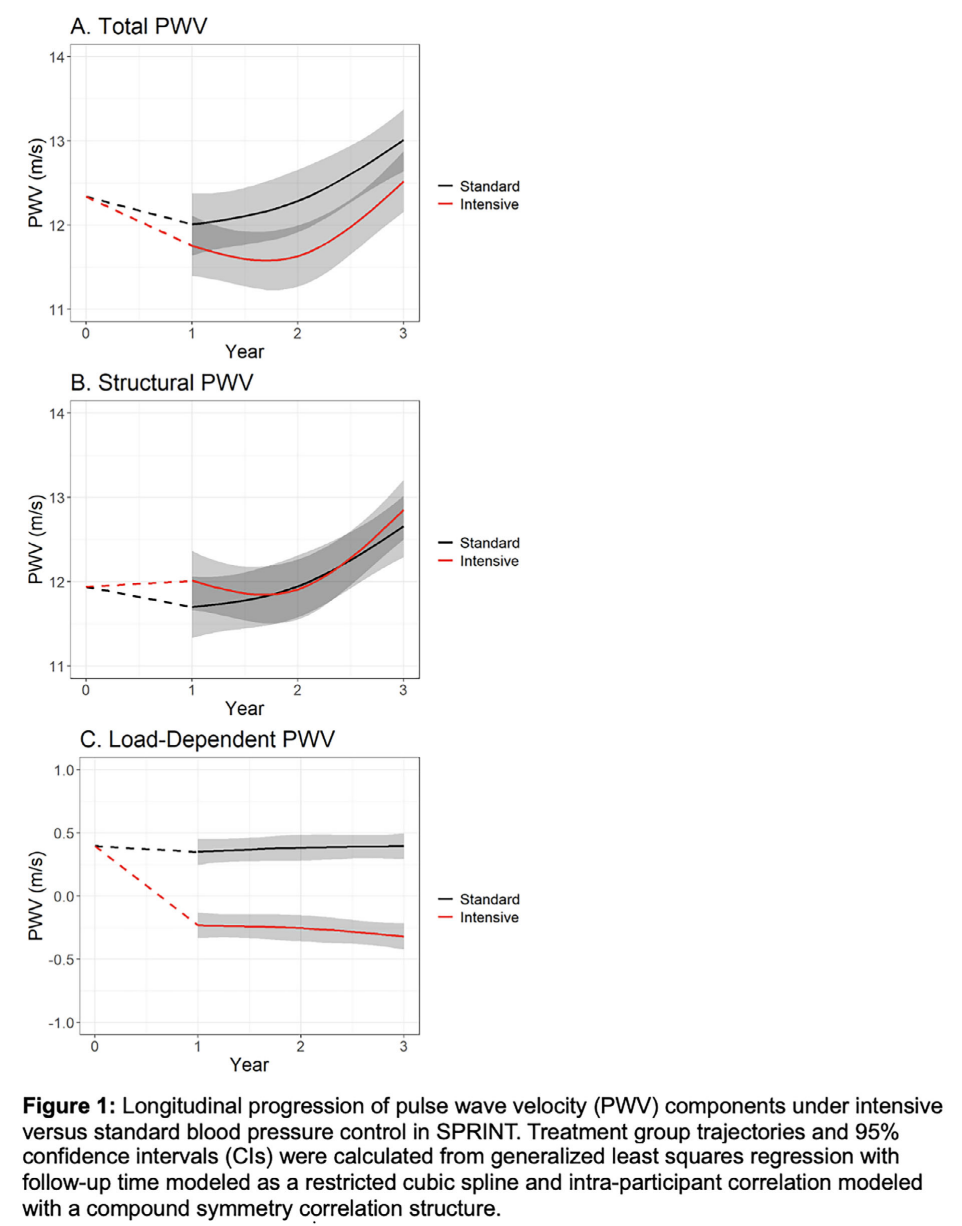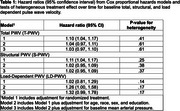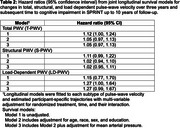# Changes in Arterial Stiffness Under Blood Pressure Control are Independently Associated with Cognitive Impairment: The Systolic Blood Pressure Intervention Trial (SPRINT)

**DOI:** 10.1002/alz70860_107420

**Published:** 2025-12-23

**Authors:** Timothy M. Hughes, Byron C Jaeger, Ryan Pewowaruk, Theodore M DeConne, Adam D Gepner, Cecilia Peterson, David M. Reboussin, Jeff Williamson, Mark A. Supiano

**Affiliations:** ^1^ Wake Forest University School of Medicine, Winston‐Salem, NC, USA; ^2^ Ryan Pewowaruk Research Consulting, Madison, WI, USA; ^3^ William S. Middleton Veteran Affairs Hospital, Madison, WI, USA; ^4^ University of Wisconsin School of Medicine and Public Health, Madison, WI, USA; ^5^ University of Utah, Salt Lake City, UT, USA

## Abstract

**Background:**

Arterial stiffness is implicated in the development of Alzheimer's disease and related dementias. The effect of intensive blood pressure control on changing arterial stiffness parameters and their associations with cognitive impairment remains unclear.

**Method:**

SPRINT evaluated the effect of intensive versus standard blood pressure control (SBP<120mmHg versus SBP<140 mmHg) on time to cognitive impairment. Arterial stiffness was assessed in a subset of participants by total carotid‐femoral pulse wave velocity (T‐PWV, in m/s) at baseline and years 1‐3. Age‐related structural stiffening (S‐PWV, due to vessel wall remodeling) and load‐dependent stiffening (LD‐PWV, due to changes in BP over the cardiac cycle) were calculated by adjusting T‐PWV to a 120/80 mmHg reference with participant‐specific models. Cognitive impairment was assessed by repeated testing over 10 years with adjudication of Mild Cognitive Impairment (MCI) or Probable Dementia (PD) as a composite outcome. Time to incident cognitive impairment was estimated as hazard ratios (HR) and 95% Confidence Intervals (95%CI) using Cox proportional hazards for baseline stiffness and Joint Longitudinal Survival Models for changes in stiffness over time adjusted for treatment arm, demographics (age, race, sex, education), baseline mean arterial pressure, and heterogeneous effects of intensive BP control.

**Result:**

A total of 611 participants (72±9 years old, 40% women, 70% White) had T‐PWV and time to event data resulting in 90 composite MCI (*n* = 62) and PD (*n* = 28) events over a median follow‐up of 6.2 years. Each m/s higher baseline T‐PWV and S‐PWV was significantly associated with a 10% increase in the hazards for developing incident cognitive impairment independent of treatment arm but was attenuated by adjustment for demographics (Table 1). LD‐PWV declined with intensive SBP control, while T‐PWV and S‐PWV increased over the 3‐year study treatment period (Figure 1). Intensive treatment‐related declines in LD‐PWV were associated a 21% reduction [HR(95%CI) = 0.79(0.61‐1.00] in the risk for cognitive impairment adjusted for demographics (Table 2). No heterogeneous treatment effects based on T‐PWV, S‐PWV, or LD‐PWV were detected.

**Conclusion:**

Components of arterial stiffness are associated with incident cognitive impairment independent of BP control. Arterial stiffness remains an unaddressed risk factor for cognitive impairment and potential target for dementia prevention.